# Leveraging Telemedicine to Spread Expertise in Neonatal Resuscitation

**DOI:** 10.3390/children9030326

**Published:** 2022-03-01

**Authors:** Joaquim M. B. Pinheiro

**Affiliations:** Department of Pediatrics, Albany Medical Center, Albany, NY 12208, USA; pinheij@amc.edu

The development and potential applications of telemedicine in neonatal resuscitation were reviewed by Donohue and colleagues in 2019, in a manuscript that compiled seminal references in the field [1]. Since then, the COVID-19 pandemic has forced profound changes in healthcare delivery. Although neonates were largely spared the direct effects of coronavirus infection, pregnant women and their newborn infants suffered from negative impacts of both the pandemic and the associated public health countermeasures which degrade socioeconomic conditions and healthcare system function; the more vulnerable sub-populations may be particularly susceptible to these effects [2]. The pandemic markedly accelerated the use of telecommunications, including telemedicine [3]. Healthcare institutions needed to develop or expand their telehealth capabilities to support patient care, as well as administrative and educational functions. This has created an opportunity to evaluate the expanded uses of telemedicine, with a particular focus on neonatal resuscitation.

Telemedicine can be applied in all aspects and phases of neonatal resuscitation. First, education and training of instructors and practitioners at remote locations can be achieved by centrally located expert instructors through teleconferencing and telesimulation. The Neonatal Resuscitation Program (NRP) [4] and Helping Babies Breathe are globally used educational programs that are designed for higher and lower-resource settings, respectively. These programs feature standardized didactic modules and simulation exercises which can be overseen by either local or off-site instructors. There is evidence that tele-education is an effective alternative to conventional training for neonatal resuscitation, resulting in comparable acquisition of knowledge and skill levels by health-care providers [5].

Then, for women with high-risk pregnancies (e.g., significant fetal anomalies), prenatal video-conferencing consultations with neonatologists and perinatologists can take place from the home setting, providing families with information that is needed to decide whether to transfer care to a regional center or continue locally. We have used this approach extensively at Albany Medical Center during the pandemic, and Lapadula et al. have reported high degrees of satisfaction from both patients and physicians with such virtual visits [3]. For women that are presenting to a remote community hospital for potential delivery of a periviable fetus (e.g., premature rupture of membranes at 21- or 22-weeks’ gestation), tele-consultation with a neonatologist and perinatologist at a regional center can similarly inform urgent decisions on the extent and location of perinatal care. This approach has the potential to diminish disparities in access of high-risk neonates to specialized care. A systematic review of early studies on the utilization of telemedicine by regional NICUs revealed promising results in the quality of care and patient satisfaction, but the conclusions were not robust and their generalizability remains unclear [6]. One regional system reported that optimizing perinatal management through telemedicine shifted high-risk, very low birthweight deliveries and consequent neonatal resuscitations towards hospitals with higher levels of NICU care, which were associated with decreased infant mortality in the region [7].

For high-risk births that are taking place in community hospitals, telemedicine can be used to support the local team during the delivery, the resuscitation, and postnatal stabilization. First, teleconsultation may help to prepare the team during the pre-resuscitation briefing. Then, in performing the basic resuscitation steps and any advanced procedures such as insertion of an artificial airway, the local team may benefit from remote expert guidance. While the use of videolaryngoscopy in neonatal intubation is still limited, the simple external visualization of the endotracheal tube depth at the lip by the remote consultant will help to avoid the common problem of endobronchial intubation, thereby improving the effectiveness and safety of ventilation [8]. The placement of a laryngeal mask airway can similarly be guided remotely through external views of the baby’s face and the airway device—including the CO_2_ detector. The recent emphasis on electrocardiographic monitoring of the heart rate also enables the remote visual assessment of the newborn’s response to resuscitation. While most publications on telemedicine-assisted neonatal resuscitation have been derived from simulations, thereby precluding conclusions on outcomes, an early study suggested that it may improve the quality of neonatal resuscitations at community hospitals, particularly for preterm newborns [9]. A recent publication demonstrated that video-assisted neonatal resuscitations improved short-term neonatal outcomes at local hospitals and decreased the need for transfers to the regional NICU in a geographically large perinatal system [10].

In the phase of post-resuscitation stabilization and evaluation, the visual and auditory examination of the newborn, as well as viewing any available physiologic monitoring trends, can be invaluable to the neonatologist at the regional center. For the review of X-rays or other imaging, direct remote access to the local picture archiving and communication system (PACS) images and tools is ideal. The information that is obtained at this stage is particularly useful to help triage neonates with respiratory symptoms, as well as those at risk for hypoxic-ischemic encephalopathy, in whom a timely neurologic assessment is critical if therapeutic hypothermia is indicated, as it should be instituted earlier than 6 h of age [11]. Conversely, neonates whose condition is reassuring can remain under the care of local practitioners and, most importantly, avoid unnecessary transports to the regional center, separation from their families, transport risks, and the added healthcare costs. During this phase, telemedicine can similarly augment the ability of a neonatologist at the regional center to provide oversight to a transport team. In the rare cases where an infant is moribund and not transferred, remote videoconferencing with a neonatologist may provide crucial support the family, local caregivers, and transport team, both in medical decision-making and psychologically.

Finally, as emphasized in the NRP, debriefing after neonatal resuscitations is a key element of process improvement for resuscitating teams [4]. The feasibility and effectiveness of remotely-facilitated debriefing has been demonstrated in a simulated environment [12]. During the pandemic, our regional perinatal center has also used teleconferencing to review and debrief selected cases of newborn resuscitation with teams at referring hospitals. These virtual debriefs are timelier, and thus potentially more effective, than face-to-face meetings which are only possible annually in a large perinatal region; they also avoid travel time, costs, and weather-related barriers. As some hospitals implement video recording of neonatal resuscitations [13,14], debriefings can be further enhanced by the joint review of relevant video clips.

The increased availability of telecommunication tools plus the social distancing that was imposed by the pandemic have accelerated the application of telemedicine in all phases of neonatal resuscitation. Where dedicated telemedicine equipment is unavailable, the widespread use of mobile devices and secure software applications can enable the basic functions of telemedicine, even in low-resource settings [15]. It is likely that needs arising from shortages of workforce numbers or expertise in neonatal care [16] will drive user-generated innovations and development of multiple models of neonatal telehealth. As these programs are implemented, it is important that they are formally evaluated on the quality of care that is provided, outcomes and cost, while considering the perspective of all stakeholders that are involved [17].
